# Posterior Reversible Encephalopathy Syndrome Associated With Severe Proton Pump Inhibitor-Induced Hypomagnesemia: A Rare Case

**DOI:** 10.7759/cureus.114348

**Published:** 2026-08-11

**Authors:** Clotilde Ciampa, Antonella Antenora, Ciro Salzano, Valentina Apuzzi, Vincenzo Bassi

**Affiliations:** 1 Neurology, San Giovanni Bosco Hospital, Local Health Authority (ASL) Napoli 1 Centro, Naples, ITA; 2 Internal Medicine, San Giovanni Bosco Hospital, Local Health Authority (ASL) Napoli 1 Centro, Naples, ITA

**Keywords:** endothelial dysfunction, esomeprazole, hypomagnesaemia, posterior reversible encephalopathy syndrome (pres), proton pump inhibitors (ppi), vasogenic oedema

## Abstract

Posterior reversible encephalopathy syndrome (PRES) is a potentially reversible cause of acute neurological deterioration. Hypertension is the most common trigger, but metabolic and drug-related causes must also be considered. We describe a patient who developed PRES with posterior circulation involvement in the absence of hypertension. The patient showed severe hypomagnesemia after prolonged proton pump inhibitor (PPI) use, where the onset of neurological symptoms occurred after the increase of esomeprazole (40 mg/day). Brain magnetic resonance imaging (MRI) showed PRES findings in the cerebellum, evolving into a gliotic lesion.

Hypomagnesemia may promote endothelial dysfunction and vasogenic edema and has been increasingly linked to PRES. In patients with PRES and no clear trigger, serum magnesium should be monitored, especially in long-term PPI users. This case highlights a reversible and preventable cause of PRES, supporting the need to monitor serum magnesium and prescribe PPIs with caution in clinical practice.

## Introduction

Magnesium is a key intracellular cation involved in multiple enzymatic processes and the regulation of vascular tone by its action as a physiological calcium antagonist [1]. Imbalances in magnesium homeostasis can impair endothelial function, affecting vascular tone and function, as well as the maintenance of endothelial integrity [2]. Among the plausible pathogenic mechanisms underlying posterior reversible encephalopathy syndrome (PRES), cerebral endothelial dysfunction has been hypothesized [3]. This endothelial dysfunction, potentially exacerbated by hypomagnesemia, could lead to dysregulation of cerebral arteriolar tone with consequent vasoconstriction or the development of vasogenic edema [3].

Hypomagnesemia may occur in several clinical settings, including gastrointestinal or renal losses, malnutrition, chronic alcoholism, and drug exposure. Proton pump inhibitors (PPIs) are among the most commonly prescribed medications worldwide and are a well-recognized but frequently overlooked cause of hypomagnesemia, particularly during long-term or high-dose therapy. Furthermore, PPIs are widely prescribed, but routine laboratory monitoring of serum magnesium is not consistently performed [4].

Posterior reversible encephalopathy syndrome (PRES) is a rare and heterogeneous neurological condition with diverse clinical and neuroradiological manifestations and multiple established etiologies as follows: hypertensive emergencies, preeclampsia/eclampsia, acute or chronic kidney failure, sepsis, autoimmune diseases (systemic lupus erythematosus {SLE}, vasculitis, scleroderma, dermatomyositis), organ transplants, medications (immunosuppressants, anti-vascular endothelial growth factor {VEGF} agents) [5].

PRES symptoms have also been reported in association with hypomagnesemia [5]. Here, we report an unusual case of PRES associated with severe, PPI-induced hypomagnesemia to stress the importance of recognizing this reversible and preventable risk factor in neurological practice.

## Case presentation

We present a case of a 72-year-old Caucasian man who started therapy with acetylsalicylic acid and esomeprazole (20 mg once daily) in 2015 following a myocardial infarction. The patient had no history of hypertension or diabetes mellitus, normal renal function, and was a nonsmoker. His only cardiovascular risk factor was dyslipidemia, which was well controlled with ezetimibe therapy.

From 2015 to 2023, serum magnesium levels were not tested, but the patient showed no clinical symptoms. In October 2023, the patient started treatment with deflazacort due to the onset of diffuse arthritis, with an increase in the esomeprazole dose to 40 mg once daily. In December, the patient was experiencing symptoms such as loss of appetite, asthenia, and postural instability that progressively worsened over several months.

In August 2024, he was admitted to the emergency room due to confusion, dizziness, nausea, vomiting, and asthenia. A neurological examination revealed cerebellar dysarthria, smooth-pursuit fragmentation, bilateral nystagmus, and postural instability with a positive Romberg sign. Blood pressure was normal. An intracranial and extracranial computed tomography (CT) angiography was normal, while a cranial CT scan showed evidence of a prevalent right cerebellar edema of undetermined nature.

**Figure 1:**
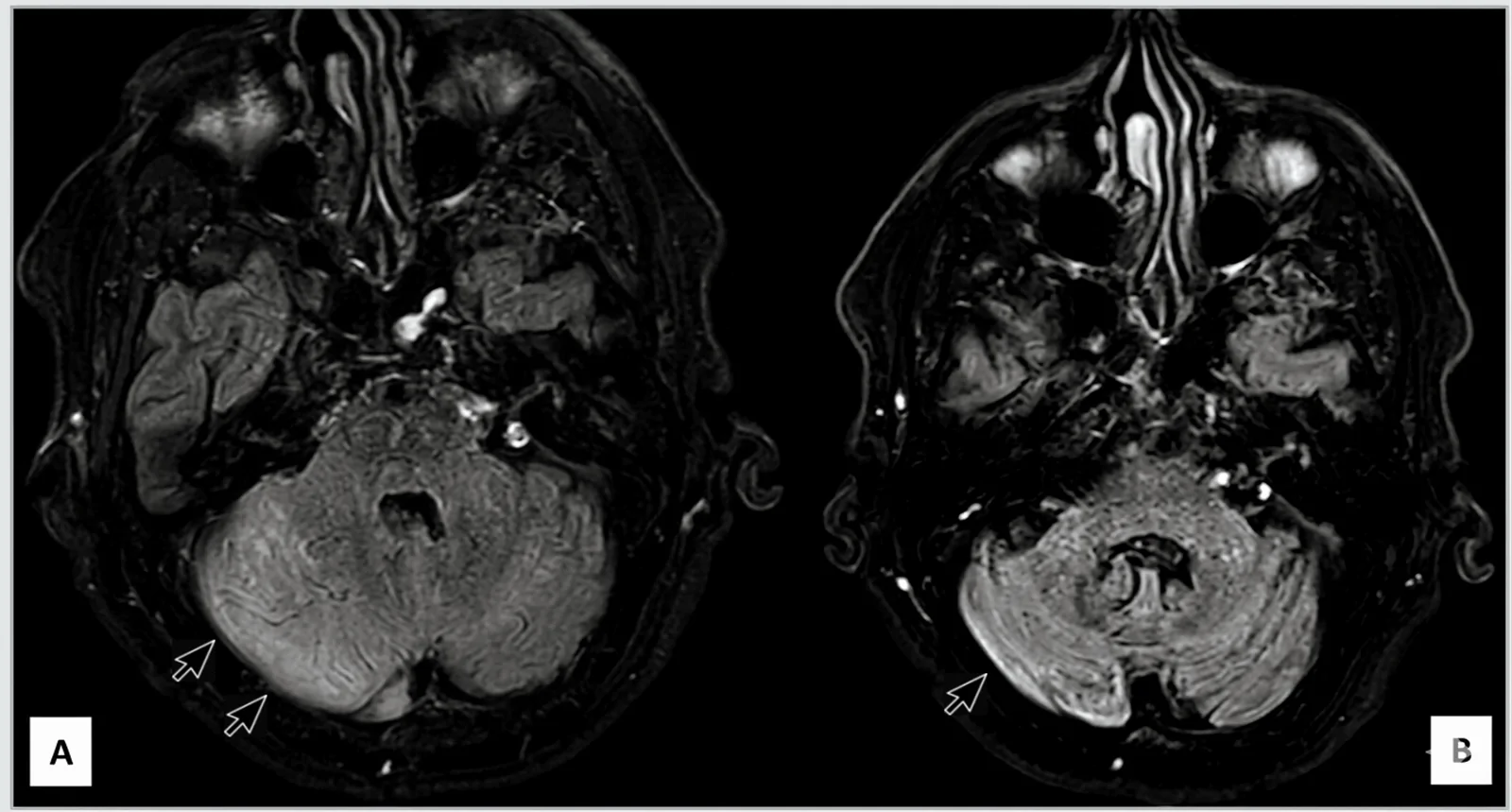
T2-FLAIR brain MRI at admission and five-month follow-up. (A) T2-FLAIR brain MRI obtained at hospital admission with blurred FLAIR hypersignal and slight T2 hypersignal in the right cerebellar hemispheric cortico-subcortical region, compatible with vasogenic edema. (B) T2-FLAIR brain MRI performed during follow-up, carried out after five months, showed a gliotic lesion in the right cerebellar hemisphere. The arrowheads indicate the area of vasogenic edema on the baseline MRI (A) and the residual gliotic lesion on the five-month follow-up MRI (B). FLAIR: fluid-attenuated inversion recovery

Brain MRI was performed using a standard neuroradiological stroke protocol, including diffusion-weighted imaging (DWI) with apparent diffusion coefficient (ADC) maps, fluid-attenuated inversion recovery (FLAIR), and susceptibility-weighted imaging (SWI). DWI demonstrated no areas of restricted diffusion, and ADC maps showed no reduction in diffusivity, excluding acute ischemic injury. SWI revealed no evidence of intracranial hemorrhage or abnormal susceptibility signal. FLAIR and T2-weighted images showed blurred fluid-attenuated inversion recovery (FLAIR) signal, hypersignal, and slight T2 hypersignal in the right cerebellar hemispheric cortico-subcortical region compatible with vasogenic edema (Figures 1A, 1B).

Contrast-enhanced imaging and MR spectroscopy were also performed. No contrast enhancement or spectroscopic abnormalities were detected to suggest a neoplastic or inflammatory process. These findings were considered highly suggestive of PRES. Consequently, a comprehensive diagnostic evaluation was started to identify or rule out the most common etiological conditions associated with the development of PRES.

Hypertensive encephalopathy was considered unlikely, as the patient remained normotensive throughout the hospital stay and did not experience any hypertensive crises. Renal failure was ruled out based on normal renal function. There was no clinical or laboratory evidence of sepsis, systemic infection, autoimmune disease, or thrombotic microangiopathy. Furthermore, the patient had no history of organ transplantation and had not been exposed to immunosuppressive agents, cytotoxic chemotherapy, or other medications commonly associated with PRES.

Laboratory tests revealed severe hypomagnesemia (0.4 mg/dL), associated with hypokalemia and hypocalcemia. Reduced urine magnesium excretion suggested that the kidneys were compensating for reduced intestinal magnesium absorption, thus excluding renal loss as the cause of the deficiency.

A detailed diagnostic workup ruled out alternative causes of magnesium deficiency, including gastrointestinal disorders, chronic diarrhea, alcoholism, uncontrolled diabetes mellitus, and endocrine disorders. The absence of other identifiable causes, combined with the patient’s history of prolonged use of proton pump inhibitors, strongly suggested that the underlying cause was proton pump inhibitor-induced hypomagnesemia.

**Figure 2:**
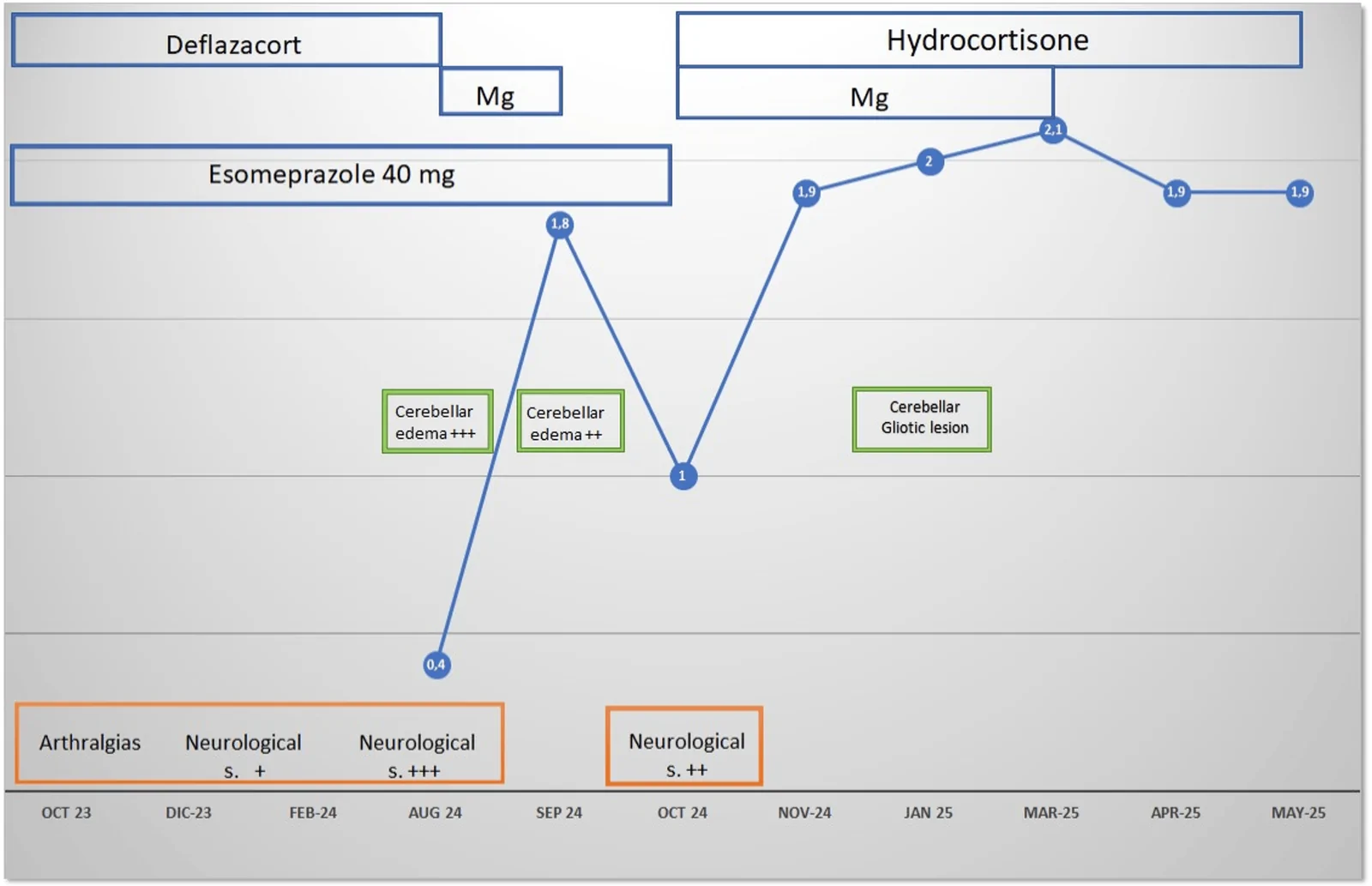
Time course of serum magnesium levels with therapies (blue boxes), MRI findings (green boxes), and clinical examinations (orange boxes).

The electrolyte abnormalities were corrected with intravenous (IV) supplementation, resulting in the normalization of serum levels and resolution of the neurological symptoms (Figure 2, Table 1). At discharge, proton pump inhibitor therapy and oral magnesium supplementation were prescribed, resulting in normal serum magnesium levels.

**Table 1:** Laboratory results changes during hospitalization, magnesium supplementation, and after discontinuation of PPI therapy. n.t.: not tested; ACTH: adrenocorticotropic hormone; t: time points of laboratory assessment; PPI: proton pump inhibitor

Variables	Admission	Discharge August 2024	Follow-up, October 2024	Follow-up, November 2024	Follow-up, March 2025	Follow-up, May 2025
Therapy	PPI oral; deflazacort	PPI; IV/oral Mg	PPI	Oral Mg; hydrocortisone	Oral Mg; hydrocortisone	Hydrocortisone
Serum Mg²⁺ (1.6-2.4 mg/dL)	0.4	1.8	1.0	1.9	2.1	1.9
Serum K⁺ (3.5-5.1 mEq/L)	2.9	4.8	4.2	4.7	4.4	4.7
Serum Ca²⁺ (8.8-10.2 mg/dL)	5.6	9.7	9.4	n.t.	n.t.	9.9
Serum Na⁺ (136-145 mEq/L)	137	138	140	139	140	139
Urine Mg²⁺ (mg/dL)	1.9	n.t.	n.t.	n.t.	n.t.	n.t.
Serum ACTH (4.7-48.8 pg/mL)	n.t.	n.t.	15	n.t.	n.t.	n.t.
Serum cortisol (4.5-24 µg/dL)	n.t.	n.t.	2.5	n.t.	n.t.	n.t.
ACTH stimulation test (serum cortisol >20 µg/dL)	n.t.	n.t.	t 0’: 7.6; t 30’: 10.7; t 60’: 12.0	n.t.	n.t.	n.t.

In October 2024, the oral magnesium supplementation was discontinued, resulting in a sudden drop in serum magnesium levels and the reappearance of neurological symptoms, such as dysarthria and postural instability. These clinical findings suggested the need to reintroduce the magnesium supplementation. Furthermore, we decided to discontinue PPI therapy to test the interference of the drug on the serum magnesium levels.

In January 2025, a brain MRI showed complete resolution of the cerebellar edema with evidence of a small gliotic sequela in the right lobe (Figures 1A, 1B). A follow-up visit revealed a normal neurological examination and normal serum magnesium levels during oral magnesium supplementation (Figure 2). In March 2025, oral magnesium supplementation was discontinued, maintaining normal serum magnesium levels (Figure 2). Additionally, hydrocortisone (30 mg twice daily) was prescribed as replacement therapy for the onset of iatrogenic hypoadrenalism secondary to previous cortisone therapy for repeated episodes of arthralgia. Currently, the patient is not taking PPIs or magnesium supplements and is maintaining normal serum magnesium levels (Table 1).

## Discussion

PRES is a clinical-radiological syndrome that was first described in 1996. Although the diagnosis is based on the clinical presentation and neuroimaging findings, there are no standardized diagnostic criteria. PRES is clinically heterogeneous and may present with headache, confusion, visual disturbances, dysarthria, ataxia, vertigo, and seizures [6,7].

Neuroimaging techniques typically reveal vasogenic edema affecting cortical and subcortical regions, though cytotoxic changes may also be present [7,8]. The frontal, parietal, and occipital lobes are the most frequently involved areas, and lesions are usually bilateral and symmetrical. However, atypical unilateral or asymmetric patterns are increasingly recognized [8,9]. The reversibility of lesions is usually common but not the rule, although long-term follow-up imaging is rarely performed [10].

In our patient, PRES presented with asymmetrical posterior circulation involvement, also in the absence of hypertension. The only identifiable trigger was severe hypomagnesemia related to prolonged use of a PPI, with symptoms emerging after the esomeprazole dose was increased to 40 mg/day. A meta-analysis of 12 studies supported a dose-dependent association between PPIs and hypomagnesemia [11].

Another unusual feature was the evolution of cerebellar edema into a gliotic lesion, indicating that PRES could lead to permanent structural damage despite apparent clinical recovery. Such sequelae are uncommon but have been reported, suggesting that follow-up imaging should be considered in selected patients [10].

This case suggests that hypomagnesemia could be a reversible cause of PRES. In clinical practice, serum magnesium levels should be checked for patients with PRES where a clear trigger is not apparent, particularly those who are long-term or high-dose PPI users. In this setting, it is advisable to reconsider PPI therapy and, where possible, use the lowest effective dose.

## Conclusions

In this study, we described an unusual clinical and neuroradiological presentation of PRES with asymmetric involvement of the posterior circulation and a residual gliotic lesion on follow-up neuroimaging. This condition was not related to a hypertensive crisis or to any other known potential causes of PRES. The only possible association was found with severe hypomagnesemia secondary to prolonged, high-dose PPI use.

Furthermore, the present case suggests a possible association between severe hypomagnesemia and PRES, supporting the hypothesis that hypomagnesemia could represent a potential etiological factor. Although this association has been explored in the literature, the available evidence remains limited to a small number of case reports and retrospective studies. Consequently, additional research is needed to better understand the etiological and pathophysiological mechanisms underlying the development of this syndrome. In conclusion, the present report suggests considering severe hypomagnesemia as a potential reversible cause of PRES, particularly in patients with unexplained PRES and receiving long-term or high-dose PPI therapy.
